# Histologic absence of yeast as a clue for classic lichen planopilaris, fibrosing alopecia in a pattern distribution, and frontal fibrosing alopecia: A cross-sectional observational study

**DOI:** 10.1016/j.jdin.2024.11.008

**Published:** 2024-12-19

**Authors:** Kimberly Williams, Antonella Tosti, Curtis T. Thompson

**Affiliations:** a Dr. Phillip Frost Department of Dermatology and Cutaneous Surgery, University of Miami Miller School of Medicine, Miami, Florida; b CTA Pathology, Beaverton, Oregon; c Departments of Dermatology and Pathology, Oregon Health and Sciences University, Portland, Oregon

**Keywords:** Alopecia, androgenetic alopecia, cicatricial alopecia, dermatopathology, female pattern hair loss, fibrosing alopecia in a pattern distribution, frontal fibrosing alopecia, hair loss, histopathology, lichen planopilaris, Malassezia, yeast

*To the Editor:*

A clinical distinction between subtle cases of primary cicatricial (scarring) alopecia, such as classic lichen planopilaris (LPP), fibrosing alopecia in a pattern distribution (FAPD), and frontal fibrosing alopecia (FFA) from noncicatricial hair loss disorders, such as female pattern hair loss (FPHL), can be difficult to make, especially when there is active seborrheic dermatitis. Both subtle LPP/FAPD/FFA and FPHL may present with pruritus, interfollicular erythema, and scaling.^1^ Similarly, a histologic distinction can be challenging in these subtle cases.^2^

Although patients with LPP/FAPD/FFA may clinically appear to have seborrheic dermatitis, we have observed that *Malassezia* spp. yeast are only rarely identified. The inflammatory clinical changes are a consequence of the lymphocyte-mediated, primary cicatricial (scarring) process in the surface, interfollicular epidermis, rather than active seborrheic dermatitis. Previous studies have reported the loss of sebaceous glands in LPP/FAPD/FFA,^2^ and, in contrast, the presence of prominent sebaceous glands (so-called pseudohyperplasia) in FPHL.^3^ The paucity of sebaceous glands in LPP/FAPD/FFA likely creates an inhospitable environment for the *Malassezia* spp. yeast. Even in early cases of LPP/FAPD/FFA, the sebaceous glands are still absent in involved follicles because the folliculocentric inflammation of the disease is always centered at the level of the infundibulum/isthmus (bulge) where the sebaceous gland is located.

The observation that *Malassezia* spp. yeast is only rarely present in LPP/FAPD/FFA is useful in distinguishing subtle cases from FPHL with associated seborrheic dermatitis. This is especially important in cases of early FFA and FAPD in which the usual perifollicular fibromyxoid scarring is not prominent and where follicular loss is minimal.^3^ In this study, we have sought to confirm the utility of this observational clue in making a histologic distinction between LPP/FAPD/FFA and FPHL with active seborrheic dermatitis.

We compared the presence of *Malassezia* spp. yeast from 137 archival biopsies (69 LPP/FAPD/FFA and 68 FPHL) from a single laboratory over a 2-year period. All patients were female (average age LPP/FAPD/FFA of 65 years and FPHL of 52 years). Definitive diagnoses of LPP, FAPD, FFA, and FPHL had been made by a dermatopathologist with hair loss expertise, who is an author of this study. Each biopsy was a single 4 mm punch, processed with transverse/horizontal sectioning, with a PAS-D stain being performed on sections that included the surface interfollicular epidermis and superficial follicular epithelium. Results were evaluated using a t-test for proportions.

There was a distinct difference between the absence and presence of yeast between cases of LPP/FAPD/FFA and FPHL. In LPP/FAPD/FFA, 98.5% (68/69) of cases had no identifiable yeast. In contrast, in FPHL 50% (34/68) of cases had identifiable yeast (*P* < .001).

This study confirms the marked difference in the absence and presence of yeast between LPP/FAPD/FFA and FPHL. This observational clue is helpful in making more definitive histopathologic diagnoses of subtle cases of both LPP/FAPD/FFA and of FPHL.

## Conflicts of interest

The authors have no conflict of interest to declare.
